# The prediction value of serum anion gap for short-term mortality in pulmonary hypertension patients with sepsis: a retrospective cohort study

**DOI:** 10.3389/fmed.2024.1499677

**Published:** 2025-01-07

**Authors:** Jinhua Zhu, Zeying Zhang, Yefei Lei, Zhenrong Ouyang, Shelby Kutty, Qiming Liu, Yunbin Xiao

**Affiliations:** ^1^Department of Pediatric Intensive Care Unit, The First People's Hospital of Chenzhou Affiliated to Jinan University, Chenzhou, China; ^2^Department of Cardiology, Xiamen Cardiovascular Hospital of Xiamen University, School of Medicine, Xiamen University, Xiamen, China; ^3^Department of Pediatric Intensive Care Unit, The First People's Hospital of Chenzhou, Chenzhou, Hunan, China; ^4^Pediatric and Congenital Cardiology, Taussig Heart Center, Johns Hopkins School of Medicine, Baltimore, MD, United States; ^5^Department of Cardiology, The Second Xiangya Hospital of Central South University, Changsha, China; ^6^Department of Cardiology, Hunan Children's Hospital Affiliated to Jinan University, Changsha, China; ^7^Department of Cardiology, The Affiliated Children's Hospital of Xiangya School of Medicine, Central South University (Hunan Children's Hospital), Changsha, China

**Keywords:** anion gap, intensive care unit, mortality, pulmonary hypertension, sepsis

## Abstract

**Background:**

The relationship between anion gap (AG) and short-term mortality of pulmonary hypertension (PH) patients with sepsis in the intensive care unit (ICU) remains unclear.

**Methods:**

This study involved a retrospective analysis of incident PH patients with sepsis first admitted to the ICU in the MIMIC IV database (2008 to 2019). Short-term outcomes include in-hospital mortality and 28-day mortality. According to the AG value (17.0 mmol/L), patients were divided into high-AG and low-AG groups. The Kaplan–Meier survival curve was used to compare the cumulative survival rates of the high and low groups using the log-rank test. Multivariable Cox regression analyses were constructed to assess the relationship between AG and short-term outcomes in PH patients with sepsis.

**Results:**

A total of 2,012 sepsis patients with PH were included. The in-hospital mortality rates (11.4%) and 28-day mortality rates (12.8%) in the high-AG group were higher than those in the low-AG group (5.0% or 7.2%, respectively; *P* < 0.001). The Kaplan–Meier curve showed that the in-hospital and 28-day cumulative survival rates were lower in the high-AG group than in the low-AG group (*P* < 0.001). The multivariable Cox regression analysis confirmed that elevated AG was an independent risk factor of in-hospital mortality, 28-day mortality, and length of stay in the ICU and hospital. The relationship between elevated AG and in-hospital mortality remains stable after subgroup analyses.

**Conclusion:**

Elevated serum AG is associated with increased risk-adjusted short-term mortality in PH patients with sepsis, and it may aid clinicians in identifying patients with poor prognosis as early as possible.

## 1 Introduction

Sepsis is a major global public health concern and one of the leading causes of death (1). Therefore, it is particularly important to determine the prognosis early and accurately. The release of cytokines and inflammatory mediators from the systemic inflammatory response caused by sepsis leads to vasoconstriction, vascular endothelial cell injury, and microthrombosis in the capillaries, which then increase pulmonary arterial pressure and induce pulmonary hypertension (PH) (2). PH is characterized by elevated mean pulmonary arterial pressure, which eventually leads to right heart failure and death (3). Patients with PH are also more susceptible to sepsis due to decreased exercise tolerance, impaired immune inflammatory system, and hypoxemia (2). The interaction between the two has led to a rapid deterioration in these patients. Concomitant PH is one of the hallmarks of poor prognosis in patients with sepsis (2, 4, 5). Therefore, close attention to the diagnosis, treatment, and prognosis of these patients is urgently needed. Although there are many indicators used to predict the prognosis of patients with sepsis, including lactate (6, 7), neuron-specific enolase (8, 9), end-tidal carbon dioxide (10), fibroblast growth factor 23 (11), and growth differentiation factor-15 (12, 13), there are still few indicators that can evaluate PH patients with sepsis. Therefore, there is still an urgent need to find simple and valid indicators to predict the prognosis of PH patients with sepsis.

Acid-base balance disorders, including metabolic acidosis, are commonly observed in the intensive care unit (ICU) and have been associated with morbidity and mortality. The anion gap (AG) can help clinicians determine the type of acid-base disease, particularly metabolic acidosis, which refers to the difference between unmeasured cations and unmeasured anions in the serum. Patients either with sepsis or with PH often have metabolic acidosis caused by elevated serum lactate levels with or without elevated AG (6, 7, 14), which suggests that AG is likely to be a prognostic indicator in PH patients with sepsis. The AG has been reported to be associated with prognosis in patients with sepsis and kidney disease (15–17), with elevated AG linked to increased mortality in ICU patients with sepsis (17). However, it is unclear whether AG can predict the clinical prognosis of PH patients with sepsis. Therefore, this study aimed to investigate the association between AG and short-term all-cause mortality in patients with sepsis and PH.

## 2 Materials and methods

### 2.1 Data source and extraction

The study data were obtained from a publicly available critical care database (Medical Information Mart for Intensive Care IV [MIMIC-IV]). One author (Jinhua Zhu), who has completed the Collaborative Institutional Training Initiative (CITI) program course (Record ID: 54790721), was approved for database access and is responsible for data extraction.

The data were abstracted from the MIMIC-IV database using the structured query language with PostgreSQL. Baseline clinical data on sex, age, ethnicity, heart rate (HR), mean arterial pressure (MAP), respiratory rate (RR), comorbidities, Charlson comorbidity index, simplified acute physiology score II (SAPS II), sequential organ failure assessment (SOFA) score, length of ICU stay, and length of hospital stay for PH patients with sepsis were collected. Comorbidities such as myocardial infarction, congestive heart failure, and peripheral vascular disease were recorded. In addition, data from blood tests including percutaneous arterial oxygen saturation (SpO_2_), white blood cell (WBC) count, platelets, hemoglobin, serum AG, sodium, potassium, total bilirubin, serum creatinine (Scr), blood urea nitrogen (BUN), troponin T, and lactate were included. The first laboratory test results were obtained after ICU admission.

### 2.2 Study population selection criteria

We retrospectively collected data from patients diagnosed with sepsis from 2008 to 2019 in the MIMIC database. Adult patients first admitted to ICU and diagnosed with sepsis were enrolled in the study. Sepsis was diagnosed based on the Sepsis-3 definition (18). Sepsis is defined as an infection combined with evidence of organ dysfunction (18). Organ dysfunction is characterized as an increased SOFA score of two points or more (18). We assumed a baseline SOFA of zero for all patients. The exclusion criteria were as follows: (1) repeated admissions; (2) missing key data; and (3) no PH.

### 2.3 Groups and endpoints

The patients were further divided into a high-AG group (*n* = 1,074) and a low-AG group (*n* = 938) based on the AG value (17.0 mmol/L). The primary endpoint was in-hospital all-cause mortality, and the secondary endpoint was 28-day all-cause mortality.

### 2.4 Statistical analysis

Continuous variables that conformed to normal distribution are expressed as mean ± standard deviation (SD), while continuous variables that did not conform to a normal distribution are presented as median (interquartile range). Continuous variables that conformed to normal distribution were analyzed using the *t*-test, while those that did not conform to normal distribution were analyzed using the Mann–Whitney U-test. Categorical data are presented as frequencies and percentages and were analyzed using the chi-square test.

The Kaplan–Meier curves were plotted, and the log-rank test was performed to compare the in-hospital and 28-day cumulative survival rates between the high-AG and low-AG groups.

Univariate Cox regression analysis was applied to determine the relationship between baseline clinical characteristics and in-hospital all-cause mortality in sepsis patients with PH. Then, multivariate Cox regression analysis was performed to determine whether elevated serum AG was independently associated with higher all-cause mortality (in-hospital and 28-day) and length of stay (in hospital and in ICU) in sepsis patients with PH. The analysis results were expressed as hazard ratios (HR) with 95% confidence intervals (CI). In model I, there were no adjustments for covariates. In model II, the following covariates were adjusted: age, sex, and ethnicity. In model III, the HR, MAP, RR, and SpO_2_ were adjusted for. In model IV of the multivariate Cox regression analysis between increased serum AG and all-cause mortality, the following covariates were adjusted: WBC, platelet, total bilirubin, troponin T, and lactate. In model IV of the multivariate Cox regression analysis examining the relationship between increased serum AG and all-cause mortality, adjustments were made for the myocardial infarct, congestive heart failure, the Charlson comorbidity index, SOFA score, and SAPS II.

All data were analyzed using the statistical software packages R 4.2.2 (http://www.R-project.org, The R Foundation) and Free Statistics software version 1.8. A two-tailed *P-value* of < 0.05 was considered statistically significant.

### 2.5 Patients and public involvement

Patients and/or the public were not involved in the design, conduct, reporting, or dissemination of this research.

## 3 Results

### 3.1 Subject characteristics

**Figure 1:**
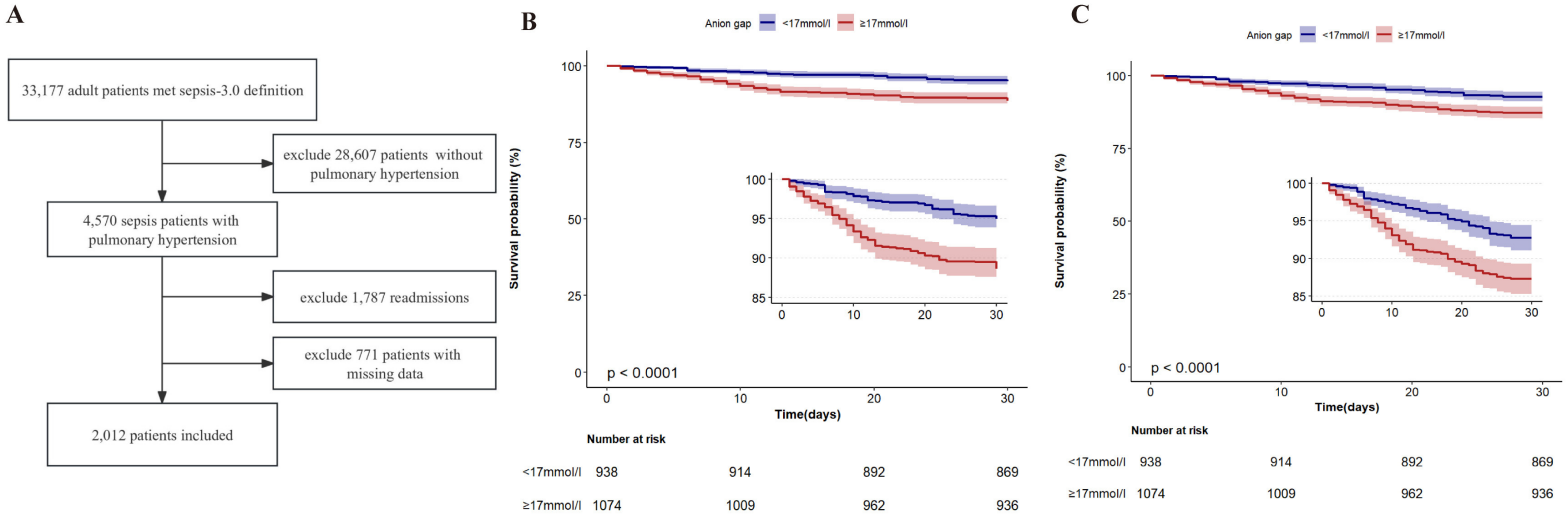
**(A)** Flowchart of the study. **(B)** Kaplan–Meier in-hospital survival estimates in the study population according to different anion gap groups. **(C)** Kaplan–Meier 28-day survival estimates in the study population according to different anion gap groups.

A total of 2,012 adult ICU PH patients with sepsis were included finally for analysis in this study, as shown in Figure 1A. The average age of the included patients was 66.2 ± 14.0 years. As shown in Table 1, compared to the low-AG group, HR (*P* < 0.001), MAP (*P* = 0.030), RR (*P* < 0.001), WBC (*P* < 0.001), platelets (*P* = 0.009), total bilirubin (*P* = 0.006), Scr (*P* < 0.001), BUN (*P* < 0.001), troponin T (*P* < 0.001), lactate (*P* < 0.001), the incidence of myocardial infarct (*P* = 0.011), congestive heart failure (*P* < 0.001), Charlson comorbidity index (*P* < 0.001), SAPS II score (*P* < 0.001), SOFA score (*P* < 0.001), length of stay in ICU (*P* = 0.003), and length of stay in hospital (*P* = 0.006) of the high-AG group were higher. However, age (*P* = 0.006), SpO_2_ (*P* = 0.024), sodium (*P* < 0.001), and proportion of white ethnicity (*P* < 0.001) in the high-AG group were lower (*P* < 0.05).

**Table 1:** Baseline characteristics of participants.

**Variables**	**Total (*n* = 2,012)**	**Low-AG group (*n* = 938)**	**High-AG group (*n* = 1,074)**	***P***
Sex, male	1,035 (51.4)	475 (50.6)	560 (52.1)	0.501
Age, years	66.2 ± 14.0	67.1 ± 13.1	65.4 ± 14.6	0.006
Ethnicity, white	1,185 (58.9)	598 (63.8)	587 (54.7)	< 0.001
Heart rate, times/min	86.0 ± 16.9	83.9 ± 14.2	87.7 ± 18.8	< 0.001
MAP, mmHg	79.9 ± 10.9	79.4 ± 9.6	80.4 ± 11.9	0.030
RR, times/min	19.9 ± 4.0	19.1 ± 3.5	20.7 ± 4.3	< 0.001
SpO_2_, %	90.2 ± 7.7	90.6 ± 7.6	89.9 ± 7.8	0.024
WBC, 10^9^/L	12.7 (9.1, 18.0)	12.4 (8.8, 16.7)	13.0 (9.2, 19.3)	< 0.001
Platelets, 10^9^/L	162.0 (107.0, 227.0)	153.0 (106.0, 212.8)	170.0 (110.0, 237.8)	0.009
Hemoglobin, g/dL	9.6 ± 2.2	9.6 ± 2.0	9.5 ± 2.3	0.188
Anion gap, mmol/L	17.5 ± 5.3	13.4 ± 2.1	21.1 ± 4.5	< 0.001
Sodium, mmol/L	136.4 ± 4.8	137.6 ± 3.6	135.4 ± 5.5	< 0.001
Potassium, mmol/L	4.0 ± 0.6	4.0 ± 0.6	4.0 ± 0.6	0.842
Total bilirubin, mmol/L	0.9 (0.5, 1.7)	0.8 (0.5, 1.6)	1.0 (0.5, 1.8)	0.026
Scr, mg/dL	1.5 (1.0, 2.4)	1.1 (0.8, 1.6)	2.0 (1.2, 3.8)	< 0.001
BUN, mg/dL	31.0 (19.0, 53.0)	23.0 (16.0, 36.0)	43.0 (26.0, 68.0)	< 0.001
Troponin T, ng/mL	0.1 (0.0, 0.4)	0.1 (0.0, 0.4)	0.1 (0.0, 0.4)	< 0.001
Lactate, mmol/L	2.1 (1.4, 3.4)	2.0 (1.4, 2.9)	2.2 (1.5, 3.9)	< 0.001
Comorbidity disease				
Myocardial infarct, n	481 (23.9)	200 (21.3)	281 (26.2)	0.011
Congestive heart failure, n	1,222 (60.7)	532 (56.7)	690 (64.2)	< 0.001
Peripheral vascular disease, n	285 (14.2)	138 (14.7)	147 (13.7)	0.511
Charlson comorbidity index	6.8 ± 2.5	6.3 ± 2.3	7.3 ± 2.6	< 0.001
SAPS II score	40.8 ± 13.1	38.0 ± 11.7	43.2 ± 13.8	< 0.001
SOFA score	4.0 (2.0, 5.0)	3.0 (2.0, 5.0)	4.0 (3.0, 6.0)	< 0.001
In-hospital mortality, n	169 (8.4)	47 (5)	122 (11.4)	< 0.001
28-day mortality, n	205 (10.2)	68 (7.2)	137 (12.8)	< 0.001
LOS in ICU, days	3.1 (1.6, 6.0)	2.8 (1.4, 5.3)	3.4 (1.8, 6.2)	0.003
LOS in hospital, days	10.0 (6.3, 16.2)	9.4 (6.1, 15.4)	10.6 (6.6, 17.7)	0.006

MAP, mean arterial pressure; Bpm, beat per minute; RR, respiratory rate; SpO_2_, percutaneous arterial oxygen saturation; WBC, white blood count; BUN, blood urea nitrogen; Scr, serum creatinine; SOFA, sequential organ failure assessment; SAPS, simplified acute physiology score; LOS, length of stay.

### 3.2 Short-term all-cause mortality

The in-hospital and 28-day all-cause mortality rates of the included patients were 8.4% and 10.2%, independently (Table 1). The in-hospital all-cause mortality rate in the high-AG group (11.4%) was significantly higher than that in the low-AG group (5.0%, *P* < 0.001), while the 28-day all-cause mortality rate in the high-AG group (12.8%) was also higher than that in the low-AG group (7.2%, *P* < 0.001), as shown in Table 1. The Kaplan–Meier curve shows that the in-hospital (log-rank test, *P* < 0.001) and 28-day cumulative survival rates (log-rank test, *P* < 0.001) were lower in the high-AG group than in the low-AG group as shown in Figures 1B, C.

### 3.3 Association between AG and short-term all-cause mortality

The univariate Cox regression showed that elevated AG was associated with in-hospital all-cause mortality (HR: 1.10, 95%CI: 1.07–1.12, *P* < 0.001). While many other clinical characteristics also increase the risk of in-hospital all-cause mortality, such as larger age (HR: 1.03, 95%CI: 1.02–1.04, *P* < 0.001), larger WBC (HR: 1.02, 95%CI: 1.01–1.03, *P* < 0.001), occurrence of myocardial infarct (HR: 1.78, 95%CI: 1.29–2.44, *P* < 0.001), congestive heart failure (HR: 1.72, 95%CI: 1.23–2.41, *P* = 0.002), elevated Charlson comorbidity index (HR: 1.15, 95%CI: 1.08–1.22, *P* < 0.001), elevated SAPS II (HR: 1.06, 95%CI: 1.05–1.07, *P* < 0.001), and elevated SOFA score (HR: 1.10, 95%CI: 1.04–1.17, *P* < 0.001), the increased SpO_2_ is associated with lower risk of in-hospital all-cause mortality (HR: 0.97, 95%CI: 0.96–0.98, *P* < 0.001). The details are shown in Table 2.

**Table 2:** Univariable analysis for in-hospital mortality.

**Item**	**HR (95%CI)**	***P***
Sex: female vs. male	0.86 (0.63, 1.16)	0.313
Age (years)	1.03 (1.02, 1.04)	< 0.001
Ethnicity: non-white vs. white	0.91 (0.67, 1.24)	0.551
Heart rate (bpm)	1.02 (1.01, 1.03)	< 0.001
MAP(mmHg)	0.97 (0.95, 0.99)	< 0.001
RR(bpm)	1.17 (1.14, 1.21)	< 0.001
SpO_2_ (%)	0.97 (0.96, 0.98)	< 0.001
WBC (× 10^9^/L)	1.02 (1.01, 1.03)	< 0.001
Platelets (× 10^9^/L)	0.96 (0.9, 1.03)	0.259
Hemoglobin (g/dl)	0.9975 (0.9958, 0.9993)	0.006
Anion gap (mmol/l)	1.1 (1.07,1.12)	< 0.001
Sodium (mmol/L)	1.03 (1,1.07)	0.06
Potassium (mmol/L)	1.0082 (0.7831, 1.298)	0.949
Total bilirubin (mmol/L)	1.0097 (1.0058, 1.0135)	< 0.001
Scr (mg/dL)	1.0028 (0.9465, 1.0625)	0.923
BUN (mg/dL)	1.05 (1.02, 1.08)	< 0.001
Troponin T (ng/ml)	1.17 (1.11, 1.24)	< 0.001
Lactate (mmol/L)	1.19 (1.14, 1.23)	< 0.001
Myocardial infarct: Yes vs. No	1.78 (1.29, 2.44)	< 0.001
Congestive heart failure: Yes vs. No	1.72 (1.23, 2.41)	0.002
Peripheral vascular disease: Yes vs. No	0.95 (0.61, 1.47)	0.808
Charlson comorbidity index	1.15 (1.08, 1.22)	< 0.001
SAPS II	1.06 (1.05, 1.07)	< 0.001
SOFA score	1.1 (1.04, 1.17)	< 0.001

MAP, mean arterial pressure; bpm, beat per minute; RR, respiratory rate; SpO_2_, percutaneous arterial oxygen saturation; WBC, white blood count; BUN, blood urea nitrogen; Scr, serum creatinine; SOFA, sequential organ failure assessment; SAPS, simplified acute physiology score.

The multivariate Cox regression showed that the high-AG (≥17.0 mmol/L) group had a higher in-hospital (HR: 2.35, 95%CI: 1.68–3.29, *P* < 0.001) and 28-day all-cause mortality (HR: 1.83, 95%CI: 1.37–2.45, *P* < 0.001). In model II, after adjusting for age, sex, and ethnicity, high AG (≥17.0 mmol/L) remained positively associated with in-hospital (HR: 2.47, 95%CI: 1.76–3.46, *P* < 0.001) and 28-day mortality (HR: 1.95, 95%CI: 1.46–2.61, *P* < 0.001). In model III, after adjusting for WBC, platelet, total bilirubin, troponin T, and lactate, high AG (≥17.0 mmol/L) remained positively associated with in-hospital and 28-day mortality (Supplementary Tables 1, 2). In model IV and model V, after adjusting for different factors, high AG was found to increase short-term all-cause mortality (Supplementary Tables 1, 2). Finally, after adjusting for all covariates mentioned above, the positive relationship between high AG and short-term all-cause mortality remained. This indicates that an increased AG (≥17.0 mmol/L) is an independent risk factor for poor outcomes in sepsis patients with PH (Supplementary Figure 1).

### 3.4 Association between AG and length of stay in hospital and ICU

The details are shown in Supplementary Tables 3, 4. The multivariate Cox regression showed that the high AG (≥17.0 mmol/L) group had a longer length of stay in hospital (β: 1.65 days, 95%CI: 0.66–2.64, *P* = 0.001) and length of stay in ICU (β: 0.69 days, 95%CI: 0.10–1.28, *P* = 0.021). In model II, after adjusting for age, sex, and ethnicity, high AG (≥17.0 mmol/L) remained positively associated with length of stay in hospital (β: 1.62 days, 95%CI: 0.63–2.62, *P* = 0.001) and length of stay in ICU (β: 0.64 days, 95%CI: 0.05–1.24, *P* = 0.033). In model III, after adjusting for WBC, platelet, total bilirubin, troponin T, and lactate, high AG (≥17.0 mmol/L) remained positively associated with length of stay in hospital (β: 1.33 days, 95%CI: 0.27–2.38, *P* = 0.014) and length of stay in ICU (β: 0.66 days, 95%CI: 0.06–1.27, *P* = 0.033).

## 4 Discussion

In the present study, we aimed to investigate the relationship between serum AG levels and recent all-cause mortality and length of stay in hospital in PH patients with sepsis. The results showed that the in-hospital mortality (*P* < 0.001) and 28-day mortality (*P* < 0.001) in the high-AG group were significantly higher than those in the low-AG group, while the length of stay in hospital (*P* = 0.001) and length of stay in ICU in the high-AG group were longer than those in the low-AG group (*P* = 0.021). The Kaplan–Meier survival curve analysis showed that the in-hospital (*P* < 0.001) and 28-day (*P* < 0.001) cumulative survival rates of the high-AG group were significantly lower than those of the low-AG group. After adjusting for covariates, the multivariate Cox regression showed that high AG (≥17.0 mmol/L) was associated with short-term all-cause mortality in patients with sepsis and PH, and this result remained stable when age was more than 60 years or in women patients or in white ethnicity patients. These results suggest that high AG (≥17.0 mmol/L) is an independent factor in predicting the short-term poor prognosis of PH patients with sepsis and can provide a basis for early intervention in these patients.

Metabolic acidosis is predisposed to occur in critically ill patients such as sepsis (19). High AG metabolic acidosis is an important subtype of metabolic acidosis, which mainly includes uremia, diabetic ketoacidosis, and lactic acidosis (20, 21). In this study, it was found that the serum lactate levels of PH patients with sepsis in the high-AG group were higher than those in the low-AG group, which may be one of the reasons for the higher AG levels in the former. The increase in serum lactate levels in PH patients may be due to increased lactate production and secretion into the peripheral blood caused by the reprogramming of pulmonary artery glucose metabolism (the Warburg effect) (22). PH patients and critically ill patients are often accompanied by hepatic dysfunction (23) and the resulting decrease in serum albumin. The concentration of serum albumin can affect the measurement of AG, which is manifested by a decrease of 0.25 mmol/L in the anion gap for every 1.0 g/L decrease in albumin. Thus, patients with hypoalbuminemia may present with normal AG when in fact they have high AG acidosis. Therefore, the reason why the AG of PH patients with sepsis in the low-AG group was lower may be due to less lactate production or hepatic insufficiency. Interestingly, in subgroup analyses, we found that the relationship between high AG and short-term mortality in PH patients with sepsis remained stable in the subgroup with serum lactate ≥2 mmol/L. However, it became negative in the subgroup with serum lactate < 2 mmol/L. This illustrates a stronger association between elevated AG and short-term mortality in PH patients with sepsis when lactate levels are high. Therefore, it is clinically important to investigate the causes of metabolic acidosis and whether AG is high (≥17.0 mmol/L) in PH patients with sepsis.

In recent years, AG has attracted the attention of clinicians as a serological indicator because of its ease of detection and calculation for the diagnosis or prognosis of various diseases. Studies have shown that elevated AG is associated with a poor prognosis, particularly in cardiovascular diseases. Serum AG, as an indicator of acidosis, may correlate with the severity or prognosis of acute myocardial infarction (AMI). Lu et al. found that elevated serum AG (≥15.12 mmol/L) is an independent predictor for short-term mortality in patients with AMI (24). Zhao et al. found that increased serum albumin-corrected AG levels are associated with increased incidence of new-onset heart failure and poor prognosis in patients with AMI (25). After albumin correction, Sheng et al. found that elevated albumin-corrected AG levels (≥20 mmol/L) are an independent risk factor for short-term and long-term mortality in critically ill patients with AMI (26). Furthermore, Zhao et al. found that AG (≥17.00 mmol/L) was an independent predictor of long-term all-cause mortality in patients after coronary artery bypass grafting and that high-AG values were associated with increased mortality (27). In addition, several studies have shown that elevated AG is also associated with an increased risk of cardiovascular or death events in conditions such as trauma (28), sepsis (17), disseminated intravascular coagulation (29), and acute pancreatitis (30). Many studies explored the relationship between AG and sepsis. Lou et al. found a negative association between elevated AG (≥18 mEq/L) at the time of ICU admission and the possibility of survival at 28 days (31). Zhou et al. examined the hypothesis in older sepsis patients and observed a significant correlation between higher albumin-corrected AG and 30-day mortality (32). Hu et al. compared the prediction value in in-hospital mortality of ICU patients with sepsis of albumin-corrected AG with AG and found that albumin-corrected AG has the highest predictive value, which is better than albumin and AG (19). In another study, Mohr et al. concluded that AG ≥20 mEq/L may be used to further risk-stratify patients for ongoing sepsis care (33). Taking into account the above studies, the association between AG and albumin-corrected AG with mortality of sepsis patients is established. However, whether the association could remain in a more severe pathophysiologic state of PH combined with sepsis which tends to cause acid-base balance disturbance is unknown. To date, no studies have investigated the relationship between mortality and AG in patients with PH. Thus, the patients included in our study were PH patients with sepsis who were hospitalized in the ICU. We explored the clinical value of different serum AG levels while assessing the predictive value of short-term outcomes. Finally, we found that high serum AG (≥17.0 mmol/L) was an independent predictor of short-term all-cause mortality in PH patients with sepsis.

There are several advantages to our study. First, the data analyzed were extracted from the MIMIC-IV database, a real-world study, which makes this study more convincing. Second, the sample size of this study was large (2,012 adult PH patients with sepsis), and the conclusions were stable. In addition, this article explores for the first time the relationship between serum AG and short-term prognosis in PH patients with sepsis, which provides a cutoff value for serum AG and can guide clinicians to assess patient prognosis and implement interventions as early as possible.

However, there are some limitations to this study. First, this study is a retrospective clinical study. Therefore, multicenter and prospective studies are needed to confirm our findings. Second, we did not conduct subgroup analyses of patients according to PH clinical groups. In addition, the endpoint of this study was short-term mortality, but the relationship between elevated serum AG and long-term mortality in PH patients with sepsis is unclear. Future studies with longer follow-ups are needed to explore the association between AG and long-term mortality in PH patients with sepsis.

## 5 Conclusion

Elevated serum AG (≥17.0 mmol/L) is associated with increased risk-adjusted short-term mortality in PH patients with sepsis, and it may remind clinicians to identify patients with poor prognosis as early as possible.

## Data Availability

Publicly available datasets were analyzed in this study. This data can be found here: Medical Information Mart for Intensive Care IV (MIMIC-IV) (https://physionet.org/).
