# Supplementary material for: The prediction value of serum anion gap for short-term mortality in pulmonary hypertension patients with sepsis: a retrospective cohort study
Source: Front Med (Lausanne). 2025 Jan 7;11:1499677. doi: 10.3389/fmed.2024.1499677 (PMC11748302; doi:10.3389/fmed.2024.1499677)
Supplement: Supplementary file 1 [file Data_Sheet_1.zip › Supplemental material/Figure S1 legend.docx]

**Figure S1.** Association between anion gap ≥ 17 mmol/L and in-hospital mortality according to baseline characteristics. Each stratification adjusted for age, sex, race, MAP, SpO_2_, Platelets, WBC, BUN, Sodium, total bilirubin, troponinT, Myocardial infarct, Congestive heart failure, Charlson comorbidity index, SOFA score, except the stratification factor itself.
