# Supplementary material for: The prediction value of serum anion gap for short-term mortality in pulmonary hypertension patients with sepsis: a retrospective cohort study
Source: Front Med (Lausanne). 2025 Jan 7;11:1499677. doi: 10.3389/fmed.2024.1499677 (PMC11748302; doi:10.3389/fmed.2024.1499677)
Supplement: Supplementary file 1 [file Data_Sheet_1.zip › Supplemental material/Table S1.docx]

| **Table S1. Association between anion gap and in-hospital mortality using an extended model approach** | | | |  |
| --- | --- | --- | --- | --- |
|  | | **Hazard ratio of**  **anion gap ≥ 17mmol/l** | **95% confidence**  **interval** | ***P* value** |
| Model 1^a^ | 2.35 | 1.68~3.29 | <0.001 |  |
| Model 2^b^ | 2.47 | 1.76~3.46 | <0.001 |  |
| Model 3^c^ | 1.73 | 1.22~2.46 | 0.002 |  |
| Model 4^d^ | 1.52 | 1.05~2.19 | 0.026 |  |
| Model 5^e^ | 1.61 | 1.13~2.29 | 0.008 |  |

^a^ *crude model.*

^b^ *adjusted for age, sex, race.*

^c^ *adjusted for HR, MAP, respiratory rate, SpO_2_.*

^d^ *adjusted for WBC, platelet, sodium, total bilirubin.*

^e^ *adjusted for Myocardial infarct, Congestive heart failure, Charlson_comorbidity_index, SOFA score.*
