# Supplementary material for: The prediction value of serum anion gap for short-term mortality in pulmonary hypertension patients with sepsis: a retrospective cohort study
Source: Front Med (Lausanne). 2025 Jan 7;11:1499677. doi: 10.3389/fmed.2024.1499677 (PMC11748302; doi:10.3389/fmed.2024.1499677)
Supplement: Supplementary file 1 [file Data_Sheet_1.zip › Supplemental material/Table S2.docx]

**Table S2. Association between anion gap and 28-day mortality using an extended model approach.**

|  | **Hazard ratio of**  **anion gap ≥ 17mmol/l** | **95%**  **confidence**  **interval** | ***P*_value** |
| --- | --- | --- | --- |
| Model 1^a^ | 1.83 | 1.37~2.45 | <0.001 |
| Model 2^b^ | 1.95 | 1.46~2.61 | <0.001 |
| Model 3^c^ | 1.40 | 1.04~1.90 | 0.028 |
| Model 4^d^ | 1.47 | 1.05~2.07 | 0.024 |
| Model 5^e^ | 1.52 | 1.13~2.05 | 0.006 |

^a^ *crude model.*

^b^ *adjusted for age, sex, race.*

^c^ *adjusted for HR, MAP, respiratory rate, SpO_2_.*

^d^ *adjusted for WBC, platelet, sodium, total bilirubin.*

^e^ *adjusted for Myocardial infarct, Congestive heart failure, Charlson_comorbidity_index, SOFA score.*
