# Supplementary material for: The prediction value of serum anion gap for short-term mortality in pulmonary hypertension patients with sepsis: a retrospective cohort study
Source: Front Med (Lausanne). 2025 Jan 7;11:1499677. doi: 10.3389/fmed.2024.1499677 (PMC11748302; doi:10.3389/fmed.2024.1499677)
Supplement: Supplementary file 1 [file Data_Sheet_1.zip › Supplemental material/Table S3.docx]

**Table S3. Association between anion gap ≥ 17mmol/l and length of stay in hospital using an extended model approach.**

|  | **β(days)** | **95%**  **confidence**  **interval** | ***P* value** |
| --- | --- | --- | --- |
| Model 1^a^ | 1.65 | (0.66~2.64) | 0.001 |
| Model 2^b^ | 1.62 | (0.63~2.62) | 0.001 |
| Model 3^c^ | 1.33 | (0.27~2.38) | 0.014 |

^a^ *crude model.*

^b^ *adjusted for age, sex, race.*

^c^ *adjusted for HR, MAP, respiratory rate, SpO2.*
