# Supplementary figures and images for: The prediction value of serum anion gap for short-term mortality in pulmonary hypertension patients with sepsis: a retrospective cohort study
Source: Front Med (Lausanne). 2025 Jan 7;11:1499677. doi: 10.3389/fmed.2024.1499677 (PMC11748302; doi:10.3389/fmed.2024.1499677)

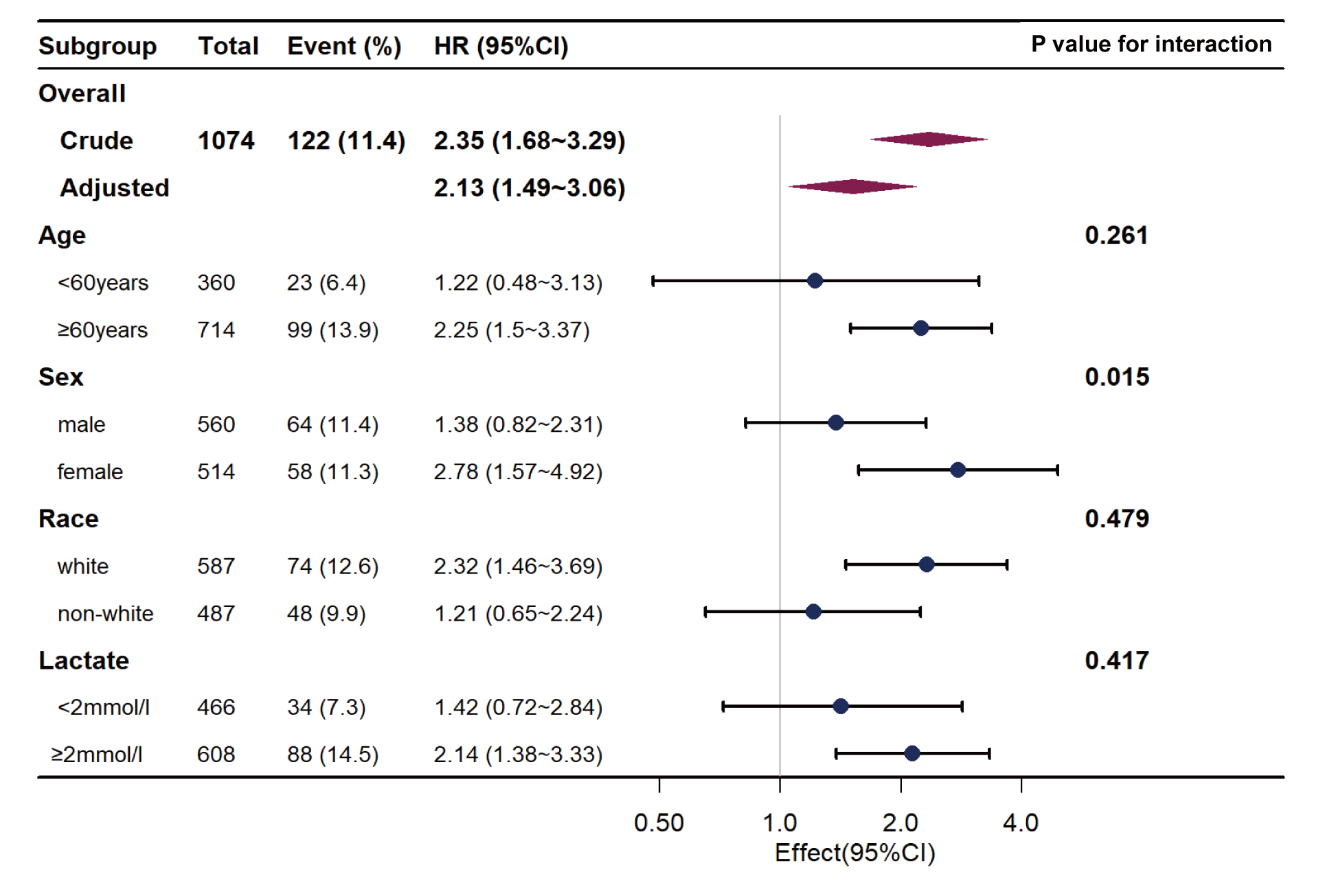

Supplement: Supplementary file 1 [file Data_Sheet_1.zip › Supplemental material/Figure S1.tif]
